# Social Media Content About Children’s Pain and Sleep: Content and Network Analysis

**DOI:** 10.2196/11193

**Published:** 2018-12-11

**Authors:** Michelle E Tougas, Christine T Chambers, Penny Corkum, Julie M Robillard, Anatoliy Gruzd, Vivian Howard, Andrea Kampen, Katelynn E Boerner, Amos S Hundert

**Affiliations:** 1 Department of Psychology and Neuroscience Dalhousie University Halifax, NS Canada; 2 Department of Pediatrics Dalhousie University Halifax, NS Canada; 3 Centre for Pediatric Pain Research IWK Health Centre Halifax, NS Canada; 4 Department of Psychiatry Dalhousie University Halifax, NS Canada; 5 Division of Neurology Department of Medicine University of British Columbia Vancouver, BC Canada; 6 Department of Mental Health BC Children’s Hospital & Research Institute Vancouver, BC Canada; 7 The Ted Rogers School of Information Technology Management Ryerson University Toronto, ON Canada; 8 School of Information Management Dalhousie University Halifax, NS Canada; 9 Department of Community Health and Epidemiology Dalhousie University Halifax, NS Canada

**Keywords:** child health, knowledge translation, pain, sleep, social media

## Abstract

**Background:**

Social media is often used for health communication and can facilitate fast information exchange. Despite its increasing use, little is known about child health information sharing and engagement over social media.

**Objective:**

The primary objectives of this study are to systematically describe the content of social media posts about child pain and sleep and identify the level of research evidence in these posts. The secondary objective is to examine user engagement with information shared over social media.

**Methods:**

Twitter, Instagram, and Facebook were searched by members of the research team over a 2-week period using a comprehensive search strategy. Codes were used to categorize the content of posts to identify the frequency of content categories shared over social media platforms. Posts were evaluated by content experts to determine the frequency of posts consistent with existing research evidence. User engagement was analyzed using Netlytic, a social network analysis program, to examine visual networks illustrating the level of user engagement.

**Results:**

From the 2-week period, nearly 1500 pain-related and 3800 sleep-related posts were identified and analyzed. Twitter was used most often to share knowledge about child pain (639/1133, 56.40% of posts), and personal experiences for child sleep (2255/3008, 75.00% of posts). For both topics, Instagram posts shared personal experiences (53/68, 78% pain; 413/478, 86.4% sleep), Facebook group posts shared personal experiences (30/49, 61% pain; 230/345, 66.7% sleep) and Facebook pages shared knowledge (68/198, 34.3% pain; 452/1026, 44.05% sleep). Across platforms, research evidence was shared in 21.96% (318/1448) of pain- and 9.16% (445/4857) of sleep-related posts; 5.38% (61/1133) of all pain posts and 2.82% (85/3008) of all sleep posts shared information inconsistent with the evidence, while the rest were absent of evidence. User interactions were indirect, with mostly one-way, rather than reciprocal conversations.

**Conclusions:**

Social media is commonly used to discuss child health, yet the majority of posts do not contain research evidence, and user engagement is primarily one-way. These findings represent an opportunity to expand engagement through open conversations with credible sources. Research and health care communities can benefit from incorporating specific information about evidence within social media posts to improve communication with the public and empower users to distinguish evidence-based content better. Together, these findings have identified potential gaps in social media communication that may be informative targets to guide future strategies for improving the translation of child health evidence over social media.

## Introduction

Social media provides fast, free, and widespread Web-based communication to millions of individuals [1]. For health communication, social media is used to share information, provide social support, connect health care professionals with the public, and inform research [2-6]. Social media research has explored several areas of child health, including cyberbullying [7], peer-support interventions [6,8], child health promotion, and intervention development [9,10].

Social media can quickly reach a variety of audiences with new information, providing easier access to evidence, and increasing the rate and breadth of engagement; for example, research shared over social media has been found to increase paper views, downloads, and impact factor [11-13]. Health care professionals report using social media for education and networking [14], with >25% using it to gather evidence and 15% for dissemination [15]. Communicating about health has been found to impact health perceptions, behaviors, and information seeking [16]. Up to 59% of parents report finding useful parenting information on social media [17], with up to 34% turning to social media to seek child health information [18]. In addition to their parents, 25% of adolescents report finding useful health information over social media [19]. Despite its popular use for sharing child health information, social media relies on open exchange and is at risk of sharing incorrect information [20,21]. With credibility as a barrier for accessing health-related social media [10,14], only 3% of parents report trusting child health information found there [18]. The level of engagement with social media for child health communication across multiple audiences has the potential to impact decision making and child health outcomes [2]. However, little is known about the content of Web-based information being shared, whether it is rooted in evidence, and the user engagement with that information.

Studies exploring health topics over social media have done so with varying methods and results, typically in adult populations, with studies seeming to focus on the content of and engagement with shared information, rather than exploring the level of evidence shared over social media. Studies have typically used content analysis over defined time periods ranging from 24 hours to 1 year, identifying a variety of content categories, and limited user engagement with health topics. Studies that explored health communication over Twitter reported that content primarily involved sharing research knowledge [22,23]. Over Facebook, where longer communication is more common, the content analysis in one study revealed posts focused less on sharing research knowledge and rather shared opportunities such as products, services, or health awareness [24]. Similarly, when searching Instagram, a photo-sharing platform that users have reported accessing to interact with others and share personal events [25], another study identified posts focused more on sharing opportunities through event promotion, advertisements, and health awareness [26]. A network analysis of Twitter posts identified low-level user engagement, within small conversation networks [27]. When exploring the level of shared research evidence, a study exploring Web-based blogs found that only 10% were evidence-based [28].

Despite the widespread use of social media, there remains little knowledge about what information about child health is actually being shared online, whether it is rooted in evidence, and the level of engagement with that information. As this information may influence child health decisions, it is imperative that Web-based conversations are studied. This research can help health care professionals and researchers understand what information is being sought after and shared. In addition, it can provide suggestions for overcoming barriers to accessing and using social media, and guide researchers and health care professionals to be credible sources [29-33] who positively influence these Web-based conversations, and help motivate evidence-based information seeking and sharing [34].

This study aims to take the first step in understanding and identifying the content of child health information publicly shared over social media, the level of evidence within that content, and user engagement with that information. To focus on this research, content areas were chosen to represent common child health topics that were likely to be frequently discussed across multiple audiences over social media—child pain and child sleep. Both child pain and sleep are problems that can impact multiple areas of functioning in a child’s life and have the potential to persist into adulthood [35,36]. Pain affects 1 in 5 children [37], and sleep problems affect approximately 25%-50% of children [38].

The primary goals are to conduct preliminary research to systematically collect, categorize, and describe the content of child pain and sleep social media posts and describe the level of shared research evidence across social media platforms. The secondary goal of this research is to examine user engagement with the child pain and sleep information shared over social media. Based on the existing research, we hypothesized that social media posts would cover a range of content categories with varying frequencies across platforms; the level of shared research evidence would be minimal, and Twitter would be used more often to share evidence, compared with Facebook and Instagram; and user engagement would be minimal and contained within small conversation networks.

## Methods

### Search Strategy

#### Development

Twitter, Instagram, and Facebook (groups and pages) were searched using pain and sleep as content areas. Based on the existing reviews about social media use, these social media platforms were selected for their popularity (percent of Web-based population using platforms), for being used across multiple age demographics, for being used most frequently (percentage of Web-based population using the platforms daily), and each using different modes for sharing information (eg, Twitter posts being 280 characters, Instagram posts sharing only images and hashtags, and Facebook sharing longer messages and images) [17,39-42].

The search strategy was iteratively developed and tested using keywords related to “child,” “pain,” and “sleep” with the aim to retrieve a sample size appropriate for analysis similar to previous studies [23,43-45], which was determined through pilot testing of the search strategy. The final search strategy was applied to the 3 social media platforms for a 2-week period (pain: December 2015, sleep: May 2016; see Multimedia Appendix 1 for each of the final strategies).

Netlytic, a cloud-based text and social networks analyzer, was used in the search process, as well as the analysis of social media posts. Netlytic automatically summarizes and discovers communication networks from publicly available social media posts [46]; it uses public Application Program Interfaces to collect posts from Twitter, Instagram, and Facebook (public groups and pages). Netlytic has been used in a number of studies exploring Web-based communities, including the motivation for healthy lifestyles [47], the impact of a Web-based reading program [48], and engagement of the Health Care Social Media of Canada community [49]. Owing to platform interface differences, the search strategy was adapted to each social media platform as outlined below.

#### Twitter

The Twitter interface permits searching simultaneously for keywords and hashtags by using Boolean operators (eg, AND, OR). Therefore, all keywords and hashtags from the final search strategy for each content area were simultaneously used to search the Twitter platform using Netlytic; this search retrieved all individual Twitter posts that included the search terms, which were subsequently screened for inclusion. Of note, individual Twitter user profiles were not searched.

#### Instagram

Instagram permits searches using only one hashtag at a time. Hashtags were placed before all “child” terms and individually searched using the Netlytic program. The child search results were imported into an excel document and filtered using a search option for posts that included any of the “sleep,” or “pain” terms from the final search strategy. Individual Instagram posts and corresponding comments were retrieved at this stage and were subsequently screened for inclusion. Of note, individual Instagram user profiles were not searched.

#### Facebook

The Facebook interface does not identify Boolean operators in the search; therefore, only 2-word searches (eg, “child pain”) were conducted at a time. Therefore, all combinations of child words (eg, child, teen, and toddler) were searched manually on the Facebook platform with each of the pain and sleep words (eg, pain, ache, and ouch). Owing to platform interface differences, Facebook cannot be searched for individual posts. Instead, the searches generated public Facebook groups and pages. Only public content was searched; therefore, individual user profiles were not explored. The lists of groups and pages generated from the search strategy were scrolled through to the bottom, until no new groups or pages were loaded by the platform. The titles of the identified groups and pages were subsequently screened according to the inclusion and exclusion criteria. Once groups and pages were identified on the basis of the inclusion criteria, all individual posts from each included group and page were retrieved using Netlytic. Each of those individual posts were subsequently screened for inclusion.

#### Inclusion Criteria

Public, English social media posts were included. Child pain posts describing acute, recurrent, or persisting pain were included as well. In addition, child sleep posts describing sleep (eg, sleep strategies) were included. “Child” included infants, children, and adolescents (age range 0-18 years).

#### Exclusion Criteria

Posts were excluded if they were non-English, ambiguous, unrelated to the population of interest, referred only to fiction, keywords only in usernames, pornographic, or linked to malware. To respect individual user privacy, and in accordance with research ethics approval, individual user profiles were not reviewed.

### Selection Process

Two trained reviewers conducted an initial screen of all posts, evaluated the inclusion criteria, and removed duplicates. Posts meeting criteria were screened again, with links followed, and titles of websites and videos considered; however, entire websites or videos were not reviewed. During the screening process, social media posts were divided among 2 independent reviewers, a Clinical Psychology PhD student and a graduate-level Research Assistant, with 20% of posts screened in duplicate and discrepancies discussed. The interrater percent agreement for the pain, and sleep searches was 85% and 90%, respectively.

### Data Extraction

The number of unique users for each search was collected, as well as the rates of retweets for posts collected over Twitter. Unique social media posts (identified following the removal of duplicates and retweets) were coded using a coding guide created through pilot testing that followed an emergent consensus process between 3 reviewers, a Clinical Psychology PhD student and 2 graduate-level Research Assistants. The reviewers independently reviewed random selections of 100 included posts, created content categories of the posts, met to discuss identified categories, and revised the classification scheme for each content category. This process was followed using randomly selected groups of 100 posts, until saturation and agreement of the final content categories were reached.

The final content categories included the following: knowledge; personal experiences; opportunities or products; news or events; and seeking information or support (Multimedia Appendix 2). Table 1 presents examples of included posts for the main 5 categories.

Using the final coding guide, only one content category was assigned to each post. If more than one category was present, the most salient theme was coded. If a reviewer was unsure of what category to apply to a post, they consulted with other reviewers to reach consensus. As this study aimed to explore the information shared specifically on social media platforms, the information was only extracted and evaluated from the post itself (including website or video titles), rather than the information that the post linked to. The interrater percent agreement for coding the pain and sleep content was 75%, and 81%, respectively.

### Data Synthesis

#### Content Analysis

The frequency of each content category was summarized for each social media platform. All duplicate and retweet posts were removed from the content analysis to avoid biasing the sample.

#### Evidence Analysis

Social media posts were compared with the evidence available in existing knowledge syntheses (eg, clinical practice guidelines and systematic reviews) identified by literature searches and research team expertise [50-63]. A senior PhD Clinical Psychology student with experience in pain and sleep research and practice evaluated and determined whether posts clearly contained evidence; contained information conflicting with, or unsupported by evidence; contained unclear information that needed consultation; or did not contain sufficient information to assess. A second PhD student with similar expertise evaluated 20% of the posts to determine the interrater reliability calculated by percent agreement, with 91% agreement for pain, and 94% agreement for sleep. Two registered Clinical Psychologists, Dr Chambers and Dr Corkum with content expertise in child pain, and child sleep, respectively, were consulted for discrepancies or uncertainties in the evidence. Table 2 presents examples of posts consistent with research evidence. Evidence within linked material was not evaluated, only the information that was presented specifically within each social media post.

#### Social Network Analysis

In the context of analyzing social media communication networks, social network analysis (SNA) is often used to study how a specific topic of interest is communicated among a group of users by examining the structure of the communication network [64-66]. Relevant to this study is SNA’s ability to measure factors such as the number of two-way conversations among users (reciprocity) and compare this with the number of one-way conversations where users may distribute information but lack any further engagement with it. In addition, SNA can measure whether groups of users are clustered, indicating whether they often communicate together about the specific topic of interest in large, or grouped around multiple disconnected or loosely connected conversations (modularity) [49]. For each communication network in the study (one per social media platform), we used Netlytic to measure both reciprocity and modularity. Unlike the content and evidence analyses, all data, including retweets and duplicated posts, were included in the analysis to explore engagement with the user-generated content. Once the network structures that display interactions among users (eg, mention, retweet, or replies) were visually examined (Multimedia Appendix 3), quantitative metrics for reciprocity and modularity were used to summarize the nature of these interactions. Specifically, the value for reciprocity is the ratio of reciprocal interactions, with values closer to 1 indicating that most users are having two-way interactions. The value for modularity is the level of network clustering, with values closer to 1 suggesting that a network consists of many weakly connected users, rather than one coherent, highly connected group [67].

**Table 1:** Examples of social media posts for each code category for the pain and sleep searches.

Categories	Pain	Sleep
Sharing information	“New research helps children suffering from chronic pain”	“#Blog: Should You be #Cosleeping with Your #Baby?”
Sharing personal experiences	“Little kiddo recovering super well from scary hand-squooshing incident. #ouch”	“My favorite fairy tale is the one where my kid actually goes to sleep after just one story”
Sharing opportunities	“This #Nursing #job might be a great fit for you: Registered Nurse- Pediatric Pain & Palliative Care”	“Using this app will help make my kid’s bedtimes easier!”
Sharing news	“Local Art competition for school kids. 15 schools. 5000 kids. Global Year Against Joint Pain”	“Where the children sleep—stunning photos show reality of life for #refugee children fleeing”
Seeking support	“Hi, I’m 14 years old and have chronic abdominal pain. I would really like someone my age to talk to who understands what pain is like”	“Any tips for getting a toddler to stay in bed gratefully received. Tearing my hair out here”

**Table 2:** Examples of social media posts sharing information consistent with research evidence for the pain and sleep searches.

Pain	Sleep
“How #breastfeeding can reduce vaccination pain in children—VIDEO:[Link]”	“Study shows that children sleep better when they have a nightly bedtime routine”
“Immersive #Virtual Reality Therapy to Control Pain during Wound Dressing Changes in Pediatric & Adult Burn Patients”	“Kids often don’t get the amount of sleep they need. Lack of sleep impacts learning. #Sleep”
“FDA recommends not using codeine for cough or pain in children”	“Bedtimes need to be set & kept. Kids need down-time & structured schedules—sleeping is part of healthy living for all”
“#mentalhealth issues are risk factors for #chronicpain in European teens”	“A chief reason for our pandemic of #teensleeplessness is that many kids nowadays unrepentantly sleep with their phones”

## Results

### Search Results

After screening and removal of duplicates, the pain search included 1133 Twitter posts by 990 users, 68 Instagram posts by 23 users, and 247 Facebook posts by 30 users from 5 groups and by 23 users from 4 pages. The sleep search resulted in substantially more posts, and a random sample was selected for analysis (20% Twitter posts, 100% Instagram posts, and 15% Facebook groups and pages). After screening and removal of duplicates, the subsample included 3008 Twitter posts by 2863 users, 478 Instagram posts by 428 users, and 1371 Facebook posts by 125 users from 31 groups and 202 users by 49 pages (Multimedia Appendix 1). The samples revealed different rates of retweets between pain- and sleep-related posts, with more retweets in the pain sample, despite a smaller overall volume of posts, with 22.15% (1133/5115) unique pain-related Twitter posts, and 71.24% (3008/4222) unique sleep-related Twitter posts after the removal of duplicates and retweets.

### Content Analysis

#### Twitter

Pain-related posts most often shared knowledge (639/1133, 56.40% of posts). Personal experiences were coded in 32.21% (363/1133) of posts with users reporting empathy, child pain, and injury. Other categories were minimally coded, including sharing opportunities or products (68/1133, 6.18%), seeking information or support (38/1133, 3.35%), and sharing news or events (25/1133, 2.20%). Unlike the pain search, sleep-related posts most often shared personal experiences (2255/3008, 75.00% of posts), describing the impact of child sleep on parents, sleep routines, or observations of children sleeping. Other coded categories were infrequently shared, including sharing opportunities or products (362/3008, 12.03%), sharing knowledge (302/3008, 10.03%), seeking information or support (55/3008, 1.83%), and sharing news or events (34/3008, 1.13%).

#### Instagram

Pain- (53/68, 78%) and sleep-related (413/478, 86.4%) posts focused on personal experiences. Sleep posts of personal experiences were most often photos of children sleeping. Pain posts infrequently shared knowledge (7/68, 10%), opportunities or products (5/68, 7%), and sought information or support (3/68, 4%). Similarly, sleep posts only occasionally shared opportunities or products (55/478, 11.5%), and knowledge (9/478, 1.9%).

#### Facebook Groups

Most pain-related posts (43/49, 88%) were generated from one chronic pain support group. Pain posts reported personal experiences (30/49, 61%) or sought information or support (14/49, 29%), with youth often reporting that seeking support was their reason for joining. Only 4% (2/49) of posts shared opportunities or products. The 31 sleep-related groups were primarily used for parents sharing personal experiences (230/345, 66.7%), discussing children’s sleep habits and management strategies. In addition, they used the groups to share opportunities or products (41/345, 11.9%), knowledge (38/345, 11.0%), and seek information or support (36/345, 10.4%).

#### Facebook Pages

The pain-related posts most often shared knowledge (68/198, 34.3%), provided organization updates (60/198, 30.3%), sought financial support (31/198, 15.7%), shared opportunities (26/198, 13.6%), and personal experiences (13/198, 6.6%). Similarly, the sleep-related pages distributed knowledge (mostly through websites, 452/1026, 44.05%), shared opportunities and products (318/1026, 30.99%), shared personal experiences (168/1026, 16.37%), or sought social support (85/1026, 8.28%).

### Evidence Analysis

#### Social Media Platforms

Across all social media platforms, child pain had a higher percentage of posts consistent with evidence (318/1448, 21.96%) compared with child sleep (445/4857, 9.16%). Evidence-based pain posts were most often pharmacological pain management (105/317, 33.1%), pain characteristics (67/317, 21.1%), information about psychological (18/317, 5.7%) and physical (18/317, 5.7%) treatments, or pain assessment (8/317, 2.5%). Evidence-based sleep posts were most often educational information (180/445, 40.4%; eg, recommended sleep duration), healthy sleep practices (161/445, 36.2%; eg, not using electronics before bedtime), and behavioral strategies (78/445, 17.5%; eg, sleep training). Child pain communication had a higher percentage of posts conflicting with existing evidence (61/1133, 5.38% of all pain posts) compared with sleep (85/3008, 2.82% of all sleep posts).

##### Twitter

From Twitter, 20.30% of pain posts (230/1133) were consistent with evidence compared with 6.75% of sleep posts (203/3008).

##### Instagram

From Instagram, 16% (11/68) of pain posts were consistent with evidence compared with 0.6% (3/478) of sleep posts.

##### Facebook

From Facebook, 20% (10/49) of pain groups and 33.8% (67/198) of pain pages were consistent with evidence compared with 6.1% (21/345) of sleep groups and 21.25% (218/1026) of sleep pages.

### Social Network Analysis

#### Twitter

For both pain and sleep, Twitter users engaged indirectly and did not reciprocate communication from one user to another, identified by minimal two-way, back-and-forth conversations; this was reflected by low reciprocity values (0.02 for pain, 0.00 for sleep) calculated by Netlytic’s SNA. The high modularity values (0.93 for pain, 0.96 for sleep) indicated that users communicating about each of these topics interacted in small groups, clustered primarily in conversations of 2 or 3 users. Lower modularity would have indicated larger groups of users interacting together, rather than the small clusters of conversations identified in the Twitter pain and sleep networks. Similar network structures formed around Instagram and Facebook posts.

#### Instagram

Both pain- and sleep-related posts had infrequent two-way conversations between users (pain, sleep reciprocity: 0.00, 0.00), most often in small clusters of users (modularity: 0.72, 0.99).

#### Facebook

Pain-related Facebook groups and sleep-related Facebook pages did not contain sufficient interactions between users to warrant analysis. The pain-related Facebook pages and sleep-related Facebook group interactions between users were minimal (pain, sleep reciprocity: 0.02, 0.00), indicating that Facebook users do not frequently reply to, or mention others by name. Although communication was generally one-sided, modularity indicated larger groups of users having conversations than identified with the other platforms, likely because they were contained user groups that liked or followed specific Facebook pages and groups (pain, sleep modularity: 0.51, 0.53).

## Discussion

### Principal Findings

#### Content Analysis

Both child pain and sleep searches revealed that social media posts cover a range of content categories. Differences in the frequency of shared content categories emerged over Twitter, with pain-related posts primarily sharing knowledge, whereas sleep-related posts were sharing personal experiences. These results suggest possible differences in communication about the 2 child health problems over Twitter, the platform that retrieved the most posts.

The final samples revealed different rates of retweets over Twitter between pain- and sleep-related posts, with more retweets in the pain sample, despite a smaller overall volume; this finding likely reflects content differences, with sleep content being more personal (thus retweeted less), and pain content being more research-based (retweeted more).

For Instagram and Facebook, the frequencies of content categories were similar across both health conditions. Instagram was primarily used for sharing personal experiences, supporting reported motivations of using Instagram to interact with others, and share personal events [25]. Facebook groups were primarily used to share personal experiences, and Facebook pages to share knowledge. Only a small sample of pain-related Facebook groups and pages were retrieved, reflecting that additional conversations may be conducted privately (eg, closed groups) and may warrant further investigation with a more publicly shared topic. Overall, the similarities between both health conditions for Instagram and Facebook platforms are unsurprising and highlight platform interface limitations and common or expected social conduct for each platform, which may be useful to inform future knowledge-sharing initiatives.

While Instagram and Facebook groups are typically used for sharing personal experiences, they are potential sources for expanding the Web-based conversation to share more knowledge across these platforms. Continuing to increase the rate of shared knowledge, while communicating the importance of being informed, may influence the social media atmosphere about child health, helping to motivate information seeking and sharing [34].

#### Evidence Analysis

The highest rates of shared evidence were found on Facebook pages, contradicting the hypothesis that Twitter would share the most evidence. Facebook pages communicate to a closed audience who has chosen to follow communication. Other sources, like Twitter or Instagram, allow for wider access to public networks. Despite 56.40% (639/1133) of Twitter pain-related posts appearing to share knowledge, only 20.30% (230/1133) of all Twitter posts were consistent with the evidence, and only 6.75% (203/3008) of sleep-related Twitter posts were evidence-based. Similar findings were identified with Instagram and Facebook analysis, with pain posts sharing a higher proportion of evidence than the sleep posts. Comparatively, the level of evidence shared about child pain and sleep is not surprising, where a study of diabetes blogs found only 10% to be evidence-based [28].

These findings highlight the potential for changing how evidence is shared over social media. Many posts that appeared to share knowledge linked externally, and the linked information was not further evaluated. In future work, instruments such as the QUEST tool [68], DISCERN [69], and Health On the Net code [70] could be used to evaluate the quality of webpage information. To address the deficit of shared evidence, posts can include short descriptions of evidence within social media posts, improving the distribution of evidence by sharing it immediately, rather than requiring users to follow a link for the actual information of interest.

#### Social Network Analysis

Both pain and sleep networks identified limited two-way conversations, displaying low-level user engagement. Network analyses of topics on sports and politics have found networks to be large with users frequently engaging in discussion [27], illustrating the potential for expanding health communication engagement. Health care professionals, organizations, and researchers are credible sources that can overcome perceived barriers of the accuracy and validity of health information on social media [29,30]. Communicating about health has been found to impact health perceptions, behaviors, and information seeking [16]. Establishing Web-based communication as a source of credible health care information allows for two-way interactions that provide opportunities to share and distribute evidence immediately. Open dialogue can be facilitated through public Facebook groups moderated by health care professionals, Twitter chats allocated to a specific topic, or Instagram posts directed to individuals.

Many health care professionals cite perceived burden, time investment, and lack of technical skills as barriers for using social media [2,14,15]. Increasing the availability of resources illustrating risks and benefits of social media engagement and providing strategies to overcome barriers can help address the associated stigma of social networking as an engagement tool [31-33,71-73].

### Limitations

This study reflected only the content of public social media posts and does not necessarily represent data shared privately (eg, closed groups and individual user profiles) across other social media sites (eg, Pinterest and YouTube) or within linked resources (eg, webpages and videos). Relative to the total number of posts on social media (>500,000 million/day), the analyzed posts on these 2 topics were only a very small portion. The retrieved posts covered a range of topics, some of which did not always fall under only one clear content area or evidence domain, potentially influencing the overall reliability of coding and classification of posts sharing evidence. Future research should consider ways to expand social media coding when one post includes multiple topics. Only 2 child health content areas were explored, child pain and sleep; future research would benefit from exploring other child health topics, such as positive health behaviors (eg, exercise), to explore communication and engagement across a wider variety of topics. Facebook groups facilitate community discussion rather than direct conversations; therefore, this type of interaction may not have been captured. Netlytic was used for data collection and SNA, which introduces limitations of functionality. For example, Instagram allows users to acknowledge posts by clicking a “like” button, a network feature that was not retrieved with this study. Finally, these data represent uncertain generalizability. Assumptions from this work can only be drawn from the 2-week data collection from unique users interacting publicly, in English, across the selected social media platforms. Although the use of the platforms addresses a wide demographic, it cannot be inferred that this is representative of all Web-based conversations about child pain or sleep.

### Conclusions

This study was a preliminary step in social media research and systematically collected and described child pain and sleep communication and engagement over social media by analyzing the shared content, level of shared research evidence, and user engagement. Twitter showed the most discrepancy in information shared, with pain topics most often sharing knowledge, and sleep topics sharing personal experiences. In contrast, Instagram and Facebook groups shared personal experiences, and Facebook pages shared knowledge. These results contribute to empirical knowledge about social media information exchange and are key to inform knowledge translation activities (eg, public health campaigns targeting general public may benefit from using a person-centered, story-telling approach on platforms like Facebook, and a more news-like approach on Twitter). While many posts claimed to link to external evidence, they failed to share evidence over social media. As such, the research and health care communities would benefit from incorporating specific information about evidence directly within social media posts, to improve communication with the public, and to empower users to distinguish the evidence-based content better. The findings that the Web-based conversation about child health is primarily one-way represent an opportunity to expand engagement through open conversations with credible sources (eg, Twitter chats with health care professionals). Together, these findings have identified potential gaps in social media communication that may be informative targets to guide future strategies for improving the translation of child health evidence over social media.

## Appendix

Multimedia Appendix 1 Pain and Sleep Social Media Search Strategies.

Multimedia Appendix 2 Pain and Sleep Content Analysis Coding Guides.

Multimedia Appendix 3 Visual Representations of Social Networks via Social Network Analysis for Twitter, Instagram, and Facebook.

## Abbreviations

SNA: social network analysis
