# Supplementary material for: Social Media Content About Children’s Pain and Sleep: Content and Network Analysis
Source: JMIR Pediatr Parent. 2018 Dec 11;1(2):e11193. doi: 10.2196/11193 (PMC6715344; doi:10.2196/11193)

Multimedia Appendix 1. Pain and Sleep Social Media Search Strategies

Final pain two-week search strategy
(child OR childs OR children OR childrens OR childhood OR kid OR kids OR daughter OR daughters OR teen OR teens OR teenager OR teenagers OR pediatric OR paediatric OR infant OR infants OR toddler OR toddlers OR newborn OR newborns OR adolescent OR adolescence OR “little guy” OR “little man”) AND (pain OR pains OR painful OR #pain OR ache OR aches OR aching OR #ache OR #ouch OR #itdoesnthavetohurt) lang:en

Final sleep two-week search strategy
(child OR childs OR children OR childrens OR childhood OR kid OR kids OR pediatric OR infant OR infants OR toddler OR toddlers OR newborn OR newborns OR adolescent OR adolescence OR “little guy” OR “little man” OR baby OR babies OR “little person”) AND (sleep OR sleeps OR sleepy OR sleeping OR asleep OR slumber OR slumbers OR slumbering OR bed OR nap OR napping OR naps OR “night terror” OR “night terrors” OR #SleepWhisperer OR #babysleep OR #sleep OR #sleepy OR #nap) lang:en

Search and screening process for the pain and sleep searches, demonstrating the number of posts included and excluded at each stage of screening.

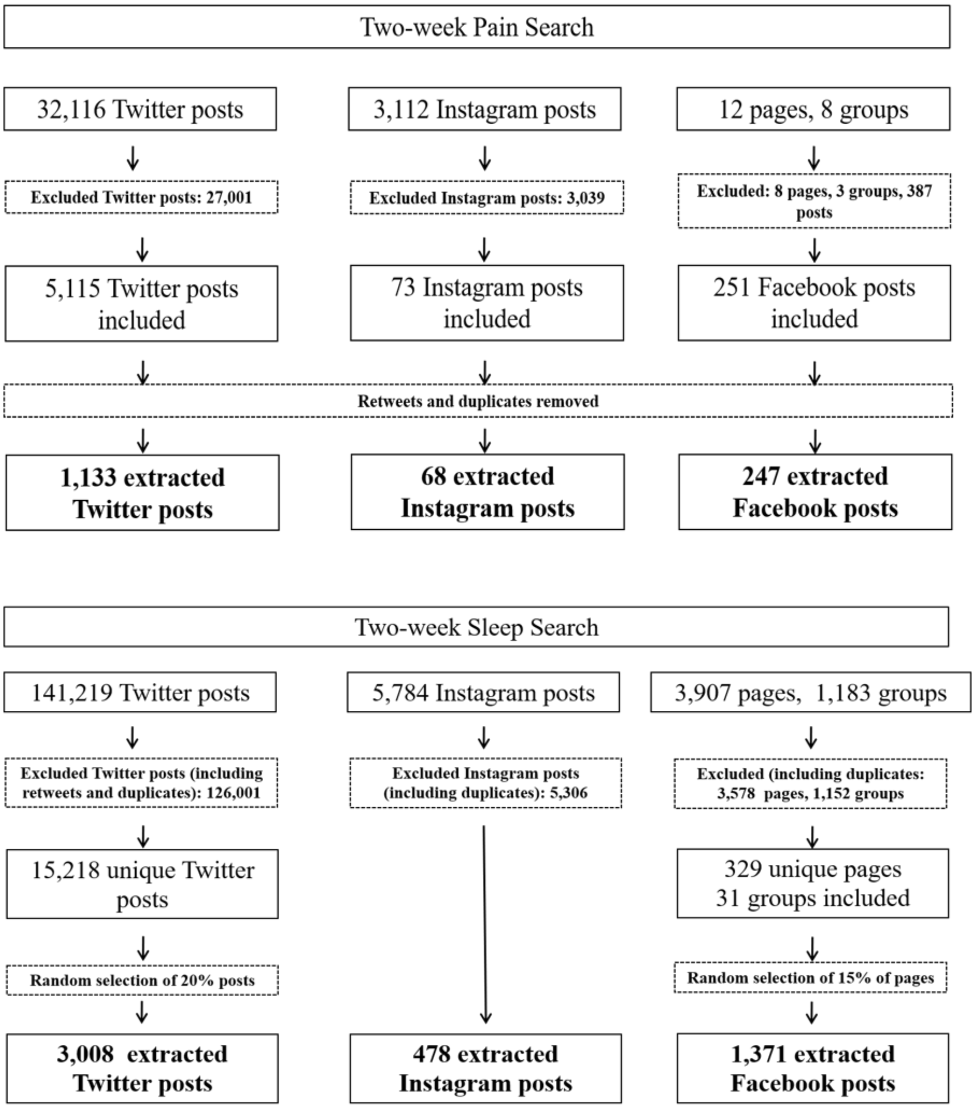

Supplement: Multimedia Appendix 1 [file pediatrics_v1i2e11193_app1.pdf]
