# Supplementary material for: Social Media Content About Children’s Pain and Sleep: Content and Network Analysis
Source: JMIR Pediatr Parent. 2018 Dec 11;1(2):e11193. doi: 10.2196/11193 (PMC6715344; doi:10.2196/11193)
Supplement: Multimedia Appendix 2 [file pediatrics_v1i2e11193_app2.pdf]

Multimedia Appendix 2. Pain and Sleep Content Analysis Coding Guides

Content Categories Identified for Coding the Pain-related Social Media Data

**Sharing knowledge**	
Resources	Web links<br>Videos<br>Images
Research findings/publications	- Trials<br>- Reviews<br>- Measurement tools<br>- Guidelines
General knowledge/information	General discussion/information
**Sharing personal experiences**	
Management strategies/treatment for child pain	
Report of child experiencing pain (general/diagnosis)	
Child injury	
Child’s shots/immunization	
Child’s response to pain	
Child’s pain relief	
Recalling childhood pain	
Caregiver response to child pain	
Consequence of pain/injury	
Appreciation of child’s support	
Providing one-on-one support to child in pain	
**Sharing opportunities/products**	
Opportunities	Funding<br>Jobs/ placements<br>Recruitment (studies/surveys)<br>Events (e.g., summer camps, training programs, support groups)<br>Webinar/Twitter chats
Advertisement/ Endorsement of product	
Sharing News/Events	
News reports/stories /blogs	
Thanks for support	
Information/update about organization/program/centre	
Promoting awareness/fundraiser	
**Sharing news/events**	
News reports/stories /blogs	
**Seeking information/support**	
Seeking financial support	Fundraising/donations
Seeking online support	Petitions/votes/likes
Seeking peer support	Someone to talk to
Seeking information	Asking advice (e.g., pain management)

Content Categories Identified for Coding the Sleep-related Social Media Data

**Sharing knowledge**	
Resources	Web links<br>Videos<br>Images
Research findings/publications	Trials<br>Reviews<br>Guidelines
General knowledge/information	General discussion/information
**Sharing opportunities/products**	
Opportunities	Funding<br>Recruitment (studies/surveys)<br>Events (e.g., camps, training programs, support groups)<br>Webinar/Twitter chats
Advertisement of product	
Thanks for support	
Information/update about organization/program/centre	
Promoting awareness/fundraiser	
**Sharing personal experiences**	
	**Recalling childhood sleep**
	**Impact of child sleep on caregivers**<br>- What parents do once child asleep<br>- Hoping child will fall/stay asleep<br>- Things done to maintain sleep<br>- Expression of relief child is sleeping<br>- Parents reaction to kids getting woken up
	**Observations about child sleeping**<br>- Report of child being asleep/ in bed<br>- Endearment about child sleeping<br>- Pictures/videos of children sleeping/in bed
	**Sleep management/habits**<br>- Bedtimes, routines<br>- Child unable to fall asleep/refusing to sleep<br>- Sleep quality (great sleep last night)<br>- Child sleeping in bed with parent<br>- Developmental transitions
	**Personal opinion:** statements about child sleep
**Sharing news/events**	
News reports/stories /blogs	
**Seeking information/support**	
Seeking financial support	Fundraising/donations
Seeking online support	Petitions/votes/likes
Seeking peer support	Someone to talk to
Seeking information	Asking advice (e.g., pain management)
