# Supplementary material for: Social Media Content About Children’s Pain and Sleep: Content and Network Analysis
Source: JMIR Pediatr Parent. 2018 Dec 11;1(2):e11193. doi: 10.2196/11193 (PMC6715344; doi:10.2196/11193)
Supplement: Multimedia Appendix 3 [file pediatrics_v1i2e11193_app3.pdf]

Multimedia Appendix 3. Visual Representations of Social Networks via Social Network Analysis for Twitter, Instagram, and Facebook

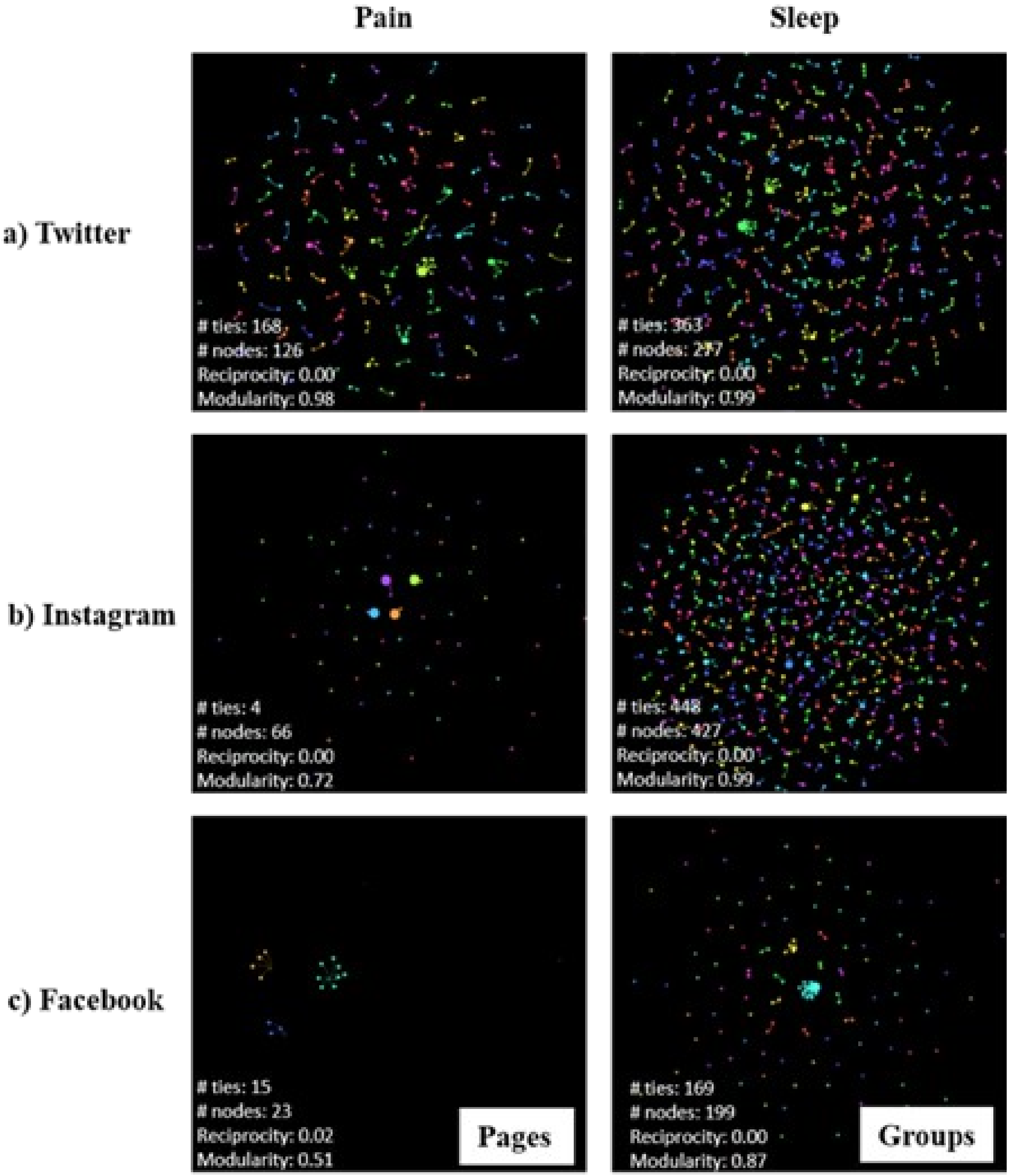

Each colored circle represents an individual user. Groups of circles of the same colors represent ‘nodes’, or groups of users communicating/sharing/reposting a social media post. Larger groups of color represent more individual users interacting with a social media post, showing higher levels of user engagement. The lines between users represent connections, or ‘ties’ between user, which may include mentioning another user in a post, or sharing a post with a specific user.
